# Impact of RAAS Receptors and Membrane-Bound Transporter System in the Left Ventricle during the Long-Term Control of Hypertension

**DOI:** 10.3390/ijms25136997

**Published:** 2024-06-26

**Authors:** Berwin Singh Swami Vetha, Rachel Byrum, DaQuan Mebane, Laxmansa C. Katwa, Azeez Aileru

**Affiliations:** 1 Department of Foundational Sciences, East Carolina School of Dental Medicine, Greenville, NC 27834, USA; byrumr19@ecualumni.ecu.edu (R.B.); mebaned16@students.ecu.edu (D.M.); 2 Department of Physiology, Brody School of Medicine, East Carolina University, Greenville, NC 27834, USA; katwal@ecu.edu

**Keywords:** angiotensin II, MAS1 proto-oncogene protein, Renin–Angiotensin–Aldosterone System, Angiotensin 1–7, (mREN2)27, angiotensin-converting enzyme, angiotensin II receptor subtype

## Abstract

The Renin–Angiotensin–Aldosterone System (RAAS) has been implicated in systemic and neurogenic hypertension. The infusion of RAAS inhibitors blunted arterial pressure and efficacy of use-dependent synaptic transmission in sympathetic ganglia. The current investigation aims to elucidate the impact of RAAS-mediated receptors on left ventricular cardiomyocytes and the role of the sarcolemma-bound carrier system in the heart of the hypertensive transgene model. A significant increase in mRNA and the protein expression for angiotensin II (AngII) receptor subtype-1 (AT_1_R) was observed in (mREN2)27 transgenic compared to the normotensive rodents. Concurrently, there was an upregulation in AT_1_R and a downregulation in the MAS1 proto-oncogene protein receptor as well as the AngII subtype-2 receptor in hypertensive rodents. There were modifications in the expressions of sarcolemma Na^+^-K^+^-ATPase, Na^+^-Ca^2+^ exchanger, and Sarcoendoplasmic Reticulum Calcium ATPase in the transgenic hypertensive model. These observations suggest chronic RAAS activation led to a shift in receptor balance favoring augmented cardiac contractility and disruption in calcium handling through modifications of membrane-bound carrier proteins and blood pressure. The study provides insight into mechanisms underlying RAAS-mediated cardiac dysfunction and highlights the potential value of targeting the protective arm of AngII in hypertension.

## 1. Introduction

Hypertension (HTN) is a condition that is prevalent in the United States and is linked to severe heart conditions, stroke, and even death [1]. It affects an estimated 47% of the U.S. population and over one billion adults globally [2]. Despite the accessibility of numerous antihypertensive drugs, achieving optimal management remains suboptimal. This highlights the necessity for a deeper understanding of the fundamental mechanisms of HTN to develop more effective therapeutic strategies.

The Renin–Angiotensin–Aldosterone System (RAAS) contributes to both systemic and neurogenic HTN. There are several available treatments for HTN. These include angiotensin-converting enzyme (ACE) inhibitors, angiotensin receptor blockers (ARBs), calcium channel blockers, diuretics, vasodilators, and beta blockers. Among these, treatment with ACE inhibitors and ARBs has been shown experimentally to be highly effective in RAAS-mediated HTN both in vivo and in vitro [3,4,5,6,7]. Angiotensin II (AngII) peptide is a key component that induces vasoconstriction and aldosterone secretion, contributing to sodium and water retention, and thereby raising blood volume and pressure [8]. Abnormal levels of AngII elevate heart rate and exert strain on the arterial walls [9]. Prolonged AngII exposure results in the thinning of these walls, complicating the expulsion of blood from the heart [10]. This can cause the left ventricle to pump more vigorously to expel blood, hence increasing the workload of the ventricular muscle [10]. However, the implications of AngII and the physiological events they instigate on cardiac function remain to be fully understood. While some studies have shown correlations between circulating plasma AngII and blood pressure in rodents, the underlying molecular mechanisms remain largely unexplored [8]. Likewise, the impact of RAAS-mediated receptors on cardiomyocytes, sarcolemma Na^+^/K^+^ ATPase (NKA), and sodium–calcium exchanger (NCX) is yet to be fully elucidated.

In this context, the (mREN2)27 transgenic rat model of HTN, characterized by the overexpression of the mouse Ren-2^d^ gene (Renin 2 tandem duplication of Ren1) in the brain and adrenal gland as well as a reduction in kidney renin, provides a valuable tool for studying the neural control of arterial pressure and cardiac excitation–contraction coupling. However, the mechanism responsible for RAAS receptor dynamics in cardiac muscle contraction in HTN is not well elucidated. The current study aims to justify the role of RAAS and receptor dynamics in the rat heart isolated from the (mREN2)27 transgenic model of HTN. Previous studies in superior cervical ganglion (SCG) concluded that the excitatory effect of AngII is mediated via AT_1_R causing a chain reaction that leads to high blood pressure [6]. Reports also show that candesartan (CandRx), an ARB, or captopril, an ACE inhibitor, has the opposite effect of AngII-induced synaptic transmission in SCG [5,7]. We hypothesize that alterations in the expression of RAAS receptors on the surface of left ventricular cardiomyocytes constitute a key mechanism involved in the signaling pathways that culminate in the manifestation of cardiac contractility and HTN. The current investigation explores receptor dynamics in the left ventricle of the heart and the role of RAAS in the ventricular contractility of the hypertensive model.

## 2. Results

### 2.1. Blood Pressure

**Figure 1:**
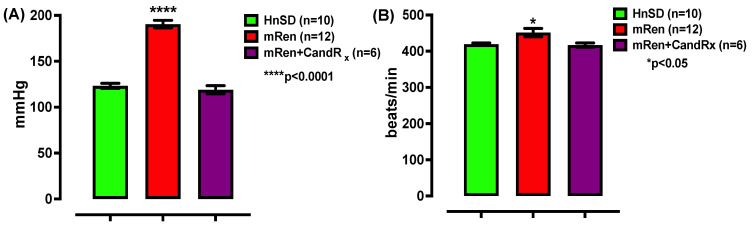
(**A**) Blood pressure measurement—systolic blood pressure was significantly higher in mRen rats compared to HnSD. Lowered blood pressure to normal, indicates AngII effects and is mediated via the AT_1_ receptor subtype. (**B**) Heart rate—resting heart rate was significantly higher in mRen rats compared to HnSD. An angiotensin receptor blocker (ARB)—candesartan (CandRx)—lowered blood pressure and the resting heart rate was normalized, indicating the effects of AngII on the rhythmicity of the heart, possibly mediated via the AT1 receptor subtype. Values are mean ± SEM (**** *p* < 0.0001, * *p* < 0.05).

Non-invasive tail-cuff plethysmography indicated a significantly elevated systolic blood pressure (SBP) and heart rate in transgenic (mREN2)27 rats compared to normotensive HnSD controls (Figure 1). Blood pressure and heart rate were measured for seven consecutive days. Two weeks of daily candesartan (16 mg/kg body weight) treatment lowered SBP and heart rate to levels comparable to HnSD controls Figure 1A,B. Longitudinal changes in the progression of systolic blood pressure and heart rate are illustrated in Supplementary Figure S1. Each data point represents an average of 10 values obtained per animal. The recordings for each day were averaged to represent the value for that day.

### 2.2. RAAS Receptor Expression and Gene Transcription Profile in Left Ventricle

**Figure 2:**
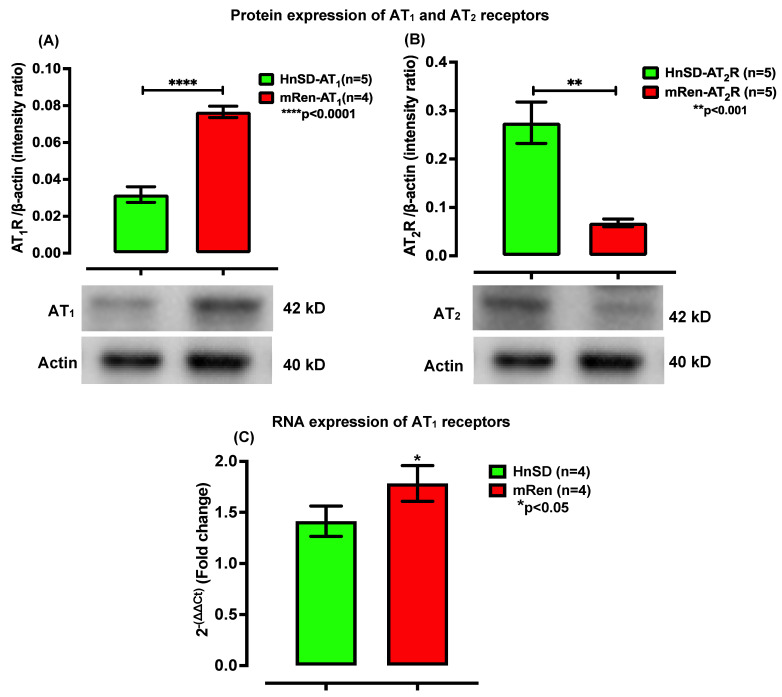
Protein expression of AT_1_R and AT_2_R receptor subtypes in left ventricle (LV) of heart. AngII receptor density was measured via Western blot hybridization using specific antibodies in LV heart isolated from (mRen2)27 hypertensive and HnSD rats. (**A**,**B**): densitometry analyses of protein level normalized to β-actin. Image analyses of the signals are normalized to β-actin. Values in each panel are means ± SEM; **** *p* < 0.0001 for AT_1_R compared to HnSD; and a diminished AT_2_R protein expression in (mRen2)27 compared to HnSD (±SEM; ** *p* < 0.001). (**C**): RNA profile expression via RT-PCR. Relative gene expression of AT_1_ mRNA profile in the left ventricle heart normalized to β-actin expression using the 2^−ΔΔCT^ comparative method and expressed as a fold change compared with HnSD. Values are means ± SEM. mRNA for AT_1_R is significantly higher in (mRen2)27 transgenic hypertensive rat heart compared with control (biological replicate of *n* = 4; HnSD 1.414, (mRen2)27 0.2, and * *p* < 0.05).

**Figure 3:**
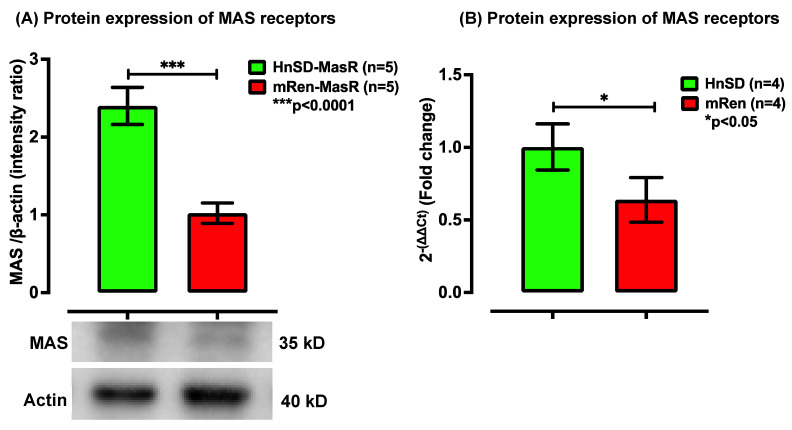
(**A**) Western blot protein expression of MAS1 receptor in left ventricle heart. Densitometry analyses of protein level normalized to β-actin. Image analyses of the signals are normalized to β-actin. Values in each panel are means ± SEM; biological replicates of *n* = 5; HnSD 2.40, (mRen2)27 1.022, and *** *p* < 0.0001. (**B**) RNA expression via RT-PCR. Relative gene expression of MAS1 mRNA profile in the left ventricle heart fold change compared with HnSD. Values are means ± SEM. mRNA for MAS1 is significantly lower in (mRen2)27 transgenic hypertensive rat heart compared with control (biological replicate of *n* = 4; HnSD 1.022, (mRen2)27 0.64, and * *p* < 0.05).

The protein expression for AT_1_R was significantly higher in left ventricular cardiomyocytes of (mREN2)27 hypertensive rats compared to HnSD (Figure 2A). The quantitative reverse transcription polymerase chain reaction (qRT-PCR) also showed a higher mRNA profile for AT_1_R (Figure 2C) in (mREN2)27 animals. Prior studies in isolated sympathetic ganglia from (mREN2)27 animals showed no significant difference in AT_1_R expression when compared to the HnSD. However, in the left ventricle, AT_1_R protein expression was abundant, presumably contributing to a driving force for cardiac output in (mREN2)27 transgenic animals. The AT_2_ G-protein-coupled (AT_2_R) receptors (Figure 2B) and the MAS1 proto-oncogene protein (Figure 3A) receptors were considerably lower in (mREN2)27. A relative gene expression for MAS1 receptors in isolated left ventricular cardiomyocytes was normalized to β-actin expression via the 2^−ΔΔCt^ comparative method and was expressed as a fold change. The result showed a diminished mRNA profile (Figure 3B) comparable to age-matched controls. The significant reduction in the mRNA profile for the MAS1 receptor as well as the decrease in AT_2_R protein expression suggests a compromise for the protective arm of AngII effects in (mREN2)27 hypertensive rodents.

### 2.3. Na^+^/K^+^ Protein Expression and Gene Transcription Profile in Left Ventricle

**Figure 4:**
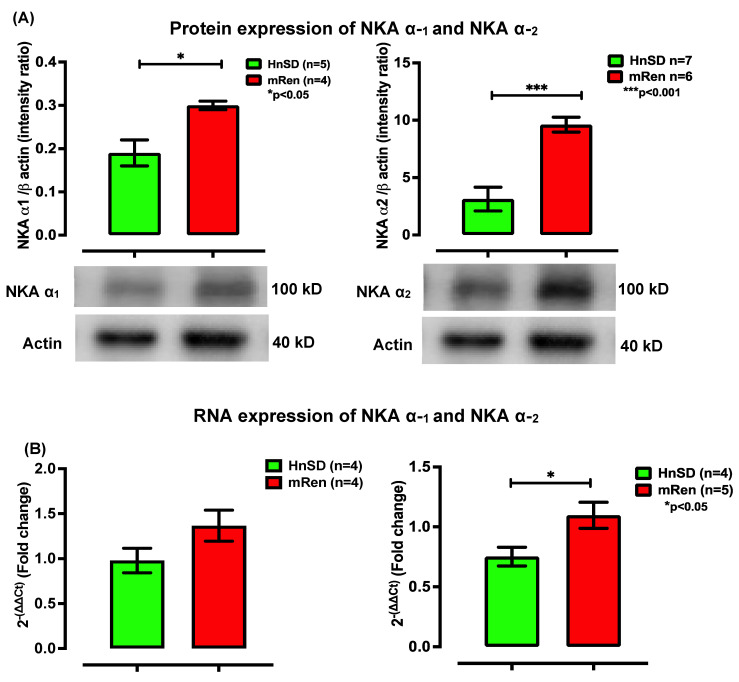
(**A**) Western blot protein expression of NKA α-1 and NKA α-2 in left ventricle heart. Densitometry analyses of protein level normalized to β-actin. Image analyses of the signals are normalized to β-actin. Values in each panel are means ± SEM; NKA α-1-HnSD 0.2, (mRen2)27 0.3, and * *p* < 0.05. NKA α-2-HnSD 3.13, (mRen2)27 9.61, and *** *p* < 0.001. (**B**) Relative gene expression of NKA α-1 and NKA α-2 mRNA profile in the left ventricle heart fold change compared with HnSD control. Values are means ± SEM. mRNA for NKA α-2 is significantly higher in (mRen2)27 transgenic hypertensive rat heart compared with HnSD (biological replicate of *n* = 4; value of 0.75 for HnSD and *n* = 5; for mRen, * *p* < 0.05).

**Figure 5:**
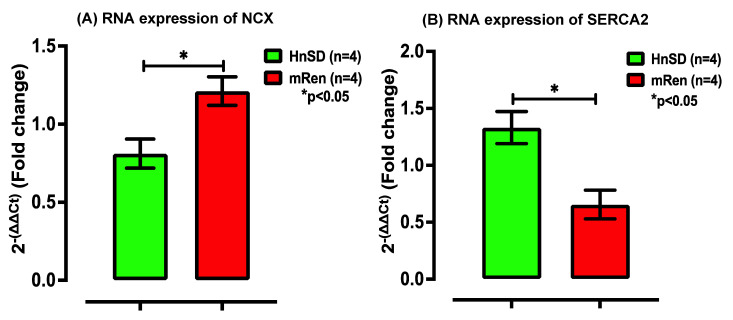
Relative gene expression of (**A**) NCX and (**B**) SERCA2 mRNA profile in the left ventricle heart fold change compared with HnSD. Values are means ± SEM. mRNA for NCX is significantly higher and SERCA2 gene expression is significantly lower in (mRen2)27 transgenic hypertensive rat heart compared with control (values in each panel are means ± SEM and biological replicate of *n* = 4; NCX-HnSD 0.811, (mRen2)27 1.212, and * *p* < 0.05. For SERCA2, HnSD 1.331, (mRen2)27 0.66, and * *p* < 0.05).

**Figure 6:**
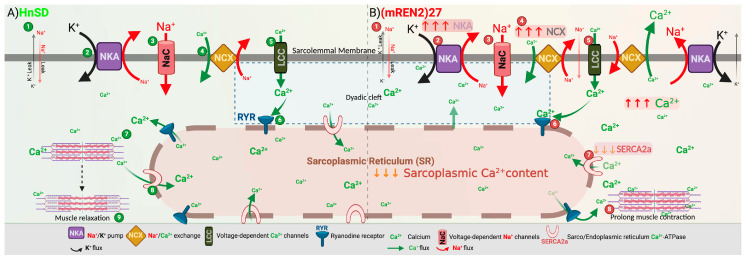
Schematic representation of [Ca^2+^]_i_ in left ventricular cardiomyocytes isolated in (mREN2)27 vs. HnSD rodents. In (mREN2)27, higher NKA isoforms (α-1 and α-2) and NCX expressions cause a surge in intracellular [Ca^2+^]_i_ influx via Ca^2+^-linked RYR receptor to deplete Ca^2+^ stores from the sarcoplasmic reticulum. However, a diminished protein expression for SERCA2 and Ca^2+^ reuptake, supporting excitation–contraction coupling and prolonged contraction in (mREN2)27 rodents. (**A**) HnSD: (1) In HnSD, sodium (Na^+^) leaks in the intracellular space, and potassium (K^+^) leaks out of the cell. (2) The Na^+^/K^+^ ATPase (NKA) pump actively pumps potassium into the cell and pumps sodium out. (3) The Na^+^ channel allows Na^+^ to go into the cell. (4) The Na^+^/Ca^2+^ exchanger (NCX) pump pumps Na+ out in exchange for the entry of Ca^2+^ into the cytoplasm. (5) Calcium ions (Ca^2+^) enter the cell through voltage-sensitive L-type Ca^2+^ channels. (6) The increase in Ca^2+^ interacts with RYR, resulting in Ca^2+^-induced Ca^2+^ release. (7) Ca^2+^ induces a conformational change in the actin and myosin filaments, resulting in the shortening of the sarcomere. (8) The SERCA2 pump facilitates the pumping back of excessive Ca^2+^ out of the cell, thereby reducing cytosolic calcium concentration. (9) The decrease in Ca^2+^ concentration helps in the restoration of sarcomere length, allowing cardiac muscle relaxation. This ensures a coordinated contraction and relaxation of the heart muscle, allowing it to maintain an optimal cardiomyocyte muscle contraction and optimal heart rate. (**B**) (mREN2)27: (1) In (mREN2)27, Na^+^ leaks into the intracellular space, and K^+^ leaks out of the cell. (2) The excessive NKA pump facilitates more K^+^ influx and Na^+^ efflux. (3) The Na^+^ channel allows Na^+^ to go into the cell. (4) The disproportionate NCX pump facilitates a rapid exchange of Na^+^ efflux for a Ca^2+^ influx into the cytoplasm. (5) Calcium ions enter the cell through voltage-sensitive L-type Ca^2+^ channels. (6) The increase in Ca^2+^ interacts with RYR, resulting in Ca^2+^-induced Ca^2+^ release. (7) Low levels of SERCA2 result in the inefficient uptake of excessive Ca^2+^, thereby increasing cytosolic calcium concentration. (8) Excessive cytosolic Ca^2+^ prolongs sarcomere length, allowing the cardiac muscle to remain constricted and thereby causing heart strain.

To determine the role of transmembrane ion transport and assess cardiac contractility during hypertensive episodes, the gene and protein expressions for Na^+^/K^+^ (NKA) transporter subunits—α_1_ and α_2_—were assessed. NKA isomers expressed protein content for the ATPase pump in the left ventricular cardiomyocytes, being normalized to β-actin and expressed as an intensity ratio compared to HnSD. Protein expression data showed similar increases in NKA isoforms during HTN when compared to control (Figure 4A). This was supported by a significantly elevated mRNA profile for NKA α_2_ in (mREN2)27 rats, while the NKA α_1_ gene profile remained unchanged (Figure 4B). Further studies are needed to elucidate the reason for this discrepancy between NKA α_1_ mRNA and protein expression. Interestingly, the expression of the NCX mRNA profile in Figure 5A showed a significant increase in (mREN2)27 rats but the relative gene expression for SERCA2 was a fold decrease in (mREN2)27 rodents compared to control animals (Figure 5B), suggesting a slow Ca^2+^ re-entry into the sarcoplasmic reticulum (SR), thereby augmenting intracellular calcium ([Ca^2+^]_i_). Figure 6 depicts a proposed schematic representation of the mechanism of action and calcium homeostasis.

## 3. Discussion

The overexpression of mouse mandibular Ren-2^d^ gene in the (mREN2)27 rats leads to the development of HTN mediated via the RAAS axis and exaggerated sympathetic nerve activity. The expression of the Ren-2^d^ gene in many tissues of (mREN2)27 hypertensive rodents has been noted, particularly in the central nervous system (CNS) and adrenal gland. It has been reported to facilitate norepinephrine release, leading to vascular resistance [11,12,13]. Previous studies have shown a correlation between mean arterial blood pressure and the exaggerated sympathetic nervous system in the presence of AngII peptide, mediated by AT_1_R [5,6]. The aim here was to understand the involvement of RAAS activation and altered cardiac function. The results show that (mREN2)27 transgenic animals have a significantly higher blood pressure and heart rate compared to normotensive animals with systolic pressure averaging ~181.2 mmHg vs. 123.8 mmHg (Figure 1A). There was a significantly higher heart rate averaging ~418.7 bpm vs. 385.4 bpm (Figure 1B). However, a daily dose of candesartan ameliorated the blood pressure and heart rate (Figure 1), suggesting that the ARB inhibits AngII mediation in hypertensive rodents. The observed normalization of cardiovascular responses with candesartan suggests a potential involvement of the angiotensin receptor system in these processes and it aligns with the known role of AngII in regulating blood pressure. Consequently, the increase in blood pressure was presumably a result of the circulating level of RAAS mediated by AT_1_R in this model of HTN.

AngII evoked responses in excitable tissues such as SCG, demonstrating RAAS behavior in the (mREN2)27 transgene model of HTN [6]. We assessed AngII-augmented excitatory receptors to understand mediation at the level of left ventricular cardiomyocytes in (mREN2)27 rodents. The antibodies for RAAS receptors were designed to recognize AT_1_, AT_2_, and MAS1 receptors from rats and mice. The peptide confirmation was verified via the amino acid analysis and mass spectrometry [14,15]. In hypertensive rodents, receptor densities for AT_1_ were about 2-fold higher than control but the AT_2_ and MAS1 receptors were one-third compared with age-matched control rodents. Furthermore, the gene transcription profile confirmed a heightened expression of AT_1_R mRNA and a significant decrease in the MAS1 proto-oncogene mRNA, which is inhibitory. Angiotensin-converting enzyme-2 (ACE2) converts AngII into Ang-(1–7) and its effect is mediated by the MAS1 receptor (Figure 3). This inhibitory component for contraction serves as the protective arm of AngII. The G-protein-coupled receptors of AT_1_ and AT_2_ generally mediate AngII peptide. While the effect of AT_1_R mediation is excitatory, AT_2_ along with Ang-(1–7)-mediated MAS1 receptors are inhibitory. The latter serve as the protective arms of RAAS at the receptor level. A lower protein expression for AT_2_, MAS1, and their gene profiles would tend to enhance the AngII excitability effect, suggesting a reduced protective capability of the receptors for myocardial excitability. Thus, a higher excitatory AT_1_R may be expected to facilitate cardiac contractility and a decrease in MAS1 proto-oncogene receptor expression as well as AT_2_R would tend to augment the contractility effects of AngII. The attenuation of MAS1 proto-oncogene receptor density faded the protection from AngII peptide, leading to the excitation of left ventricular cardiomyocytes in (mREN2)27 hypertensive rodents.

To understand the mechanism of cardiac excitation–contraction coupling and Ca^2+^ homeostasis, we assessed the membrane-bound NKA pump isoforms, NCX and intracellular sarco/endoplasmic reticulum Ca^2+^-ATPase (SERCA2), to explore the mechanism for the modulation of myocardial contraction and Ca^2+^ re-entry into the sarcoplasmic reticulum (SR). The Na^+^-lag hypothesis indicates that inhibiting myocardial membrane-bound NKA activity via cardiotonic steroids, such as ouabain or any cardiac glycoside, leads to an increase in intracellular Na^+^, which in turn leads to increased intracellular Ca^2+^ and contractility [16,17]. The results showed that there is higher protein expression for NKA isoforms during HTN (Figure 4). This was reinforced by the gene profile for NKA isoforms, suggesting an active efflux of Na ions in exchange for calcium ions to maintain resting membrane potential when compared to control. Interestingly, NCX mRNA expression was significantly high in (mREN2)27 left ventricular cardiomyocytes (Figure 5A). However, SERCA2 gene expression was downregulated in (mREN2)27 animals (Figure 5B).

These findings suggest a potential disruption in intracellular Ca^2+^ homeostasis, with increased calcium influx via NCX and potentially reduced calcium reuptake into the sarcoplasmic reticulum due to decreased SERCA2 activity and slow Ca^2+^ re-entry into SR. Taken together, the results suggest AngII-induced AT_1_R mediates sarcolemma excitability and the MAS1 proto-oncogene receptors along with AT_2_R to serve as the protective arms of AngII. The expression of membrane-bound NKA isoforms and NCX may have triggered the intracellular influx of calcium and ryanodine (RYR)-linked SR receptor for an increase in sarcoplasmic [Ca^2+^]_i_. A diminished sarcoplasmic SERCA2 calcium reuptake machinery suggests a decrease in Ca^2+^ reuptake, leading to excess [Ca^2+^]_i_ in the cytoplasm. This consequentially causes an increase in contractility and dysfunction in [Ca^2+^]_i_ homeostasis in this model of HTN. Figure 6 illustrates a hypothetical mechanism by which RAAS activation in HTN could influence ion transport and calcium homeostasis, ultimately affecting cardiac function. 

We acknowledge the importance of gender differences and biological variables in biomedical research. The use of male rats in the study is to minimize the potential variability that could arise from unknown sources in female rodents. Male (mREN2)27 transgenic hypertensive rats were studied to be able to maintain a more controlled cellular microenvironment. However, studies to explore the role of RAAS in female hypertensive models are warranted, and it would be interesting to compare any differences or lack thereof. We do acknowledge that the changes in expression do not always correlate with changes in cardiac function. Further studies should be planned to assess the functional consequences of these changes in transporter expression using electrophysiological measurements of intracellular calcium levels and contractile force. This will allow us to directly link changes in ion transport to altered cardiac function in hypertension. It would be appropriate to investigate whether these alterations in calcium handling are reversible with antihypertensive treatment.

## 4. Conclusions

This study explored the interplay between AngII signaling and cardiac dysfunction in a model of HTN induced via the Ren-2^d^ gene in (mREN2)27 rats. We aimed to understand how the chronic activation of the RAAS influences cardiac function. Collectively, our results suggest that chronic RAAS activation in (mREN2)27 HTN promotes (1) enhanced cardiac contractility through AngII-mediated AT_1_R stimulation and a compromised protective arm of AngII involving MAS1 and AT_2_ receptors and (2) disrupted calcium homeostasis due to increased NCX activity and decreased SERCA2 activity. These findings offer valuable insights into the mechanisms underlying RAAS-mediated cardiac dysfunction in HTN and highlight the potential therapeutic benefit of targeting the protective arm of the RAAS for long-term blood pressure control. Further studies are necessary to definitively establish the roles of AngII signaling pathways in cardiomyocyte dysfunction in this model. Additional studies are necessary to confirm the functional consequences of these changes in transporter expression. Electrophysiological measurements of intracellular calcium levels and contractile force would provide direct evidence linking RAAS-mediated changes in ion transport to altered cardiac function in HTN.

## 5. Perspectives

AngII facilitates cardiac excitation–contraction via AT_1_R but the low receptor dynamics for AT_2_R and MAS1 exacerbated the effects of AngII and cardiac excitability in HTN. In healthy cardiomyocytes (Figure 6A), the action potential propagates into the t-tubules, opening voltage-gated L-type Ca^2+^ channels. The resulting Ca^2+^ influx triggers the opening of Ca^2+^ release channels (ryanodine receptors) in the membrane of the sarcoplasmic reticulum (SR). The released Ca^2+^ initiates contraction as it binds to the contractile apparatus, and relaxation occurs as Ca^2+^ is recycled into the SR via SERCA2 and removed from the cell via the NCX.

Our study in the (mREN2)27 hypertensive model suggests a link between the compromised protective arm signaling of the RAAS and cardiac dysfunction (Figure 6B). We observed a decreased expression of the protective AT_2_R and MAS1 receptors, while AT_1_R expression, NKA activity, and NCX activity increased. Additionally, SERCA2 activity, crucial for calcium reuptake, was diminished. These findings highlight the potential role of the protective arm in RAAS-mediated HTN and cardiac dysfunction. The approach could offer promising avenues for managing the contributors of long-term approaches to HTN and its associated cardiovascular complications by restoring calcium homeostatic control and ameliorating cardiac dysfunction.

## 6. Materials and Methods

### 6.1. Animals Use and Candesartan Treatment

Experiments were performed in male Hannover Sprague Dawley (HnSD) and male hemizygous hypertensive (mREN2)27 transgenic rats (12–16 weeks old) obtained from the Hypertension & Vascular Research Center Colony, Wake Forest University School of Medicine, Winston-Salem, NC. The age group of the rodents was chosen because the rats represent adolescence and post-adolescence in humans [18]. Rats were housed in humidity- and temperature-controlled rooms (12-h light/dark cycle) with free access to water and standard rat chow. Animals were maintained at a fully accredited American Association for Accreditation of Laboratory Animal Care (AAALAC) unit at East Carolina University, and all experimental protocols were approved by the Institutional Animal Care and Use Committee of East Carolina Division of Health Sciences and maintained by the Department of Comparative Medicine (AUP# F100a). For the AngII receptor blocker treatment group, (mREN2)27 rats were administered candesartan 16 mg/kg body weight intraperitoneally for fourteen days.

### 6.2. Non-Invasive Blood Pressure Measurement

Indirect blood pressure and heart rate were recorded via tail-cuff plethysmography (NIBP-8; Columbus Instruments, Columbus, OH, USA). The device provides constant rates of cuff inflation and deflation over a period of 30 min. In this procedure, conscious rats were restrained in acrylic animal holders for 5–10 min in a dark, warm, quiet room, and were conditioned to numerous cuff inflation–deflation cycles. Seven-day average values for blood pressure and heart rate in each rat were subsequently obtained from eight to ten sequential cuff inflation–deflation cycles every day for one week. The onset of oscillations and the maximum amplitude observed during cuff deflation with this system were taken as the systolic and mean arterial blood pressures, respectively [5]. Total systolic, diastolic, and mean arterial pressure and heart rate recordings for 7 consecutive days were averaged for HnSD (*n* = 10), (mREN2)27 (*n* = 12), and CandRx (*n* = 6) animals.

### 6.3. Tissue Harvest

Animals were euthanized via CO_2_ asphyxiation. CO_2_ was gradually introduced into a chamber at a rate of 10–30% chamber volume per minute until respiratory arrest was achieved. The CO_2_ flow was then maintained in the chamber to ensure a complete cessation of breathing. The left ventricle of the heart was dissected for Western blotting and mRNA isolation. Samples designated for mRNA isolation were preserved in RNA later, and samples designated for Western blotting were stored at −80 °C until use.

### 6.4. RAAS Receptor Expression on the Left Ventricle: Western Blotting 

Western blots are used to visualize protein expression using chemiluminescence via antibody fluorescence. The left ventricle heart tissue is weighed, added to RIPA buffer, and homogenized on ice. Samples are allowed to sit at 4 °C to settle and then centrifuged to separate unwanted tissue that was not broken down. The supernatant was then transferred to new tubes and protein quantification was performed using Qubit protein protocol and protein broad-range assay kits (Thermo Fisher Scientific, Waltham, CA, USA). Sodium dodecyl-sulfate polyacrylamide gel electrophoresis (SDS-PAGE) was performed using 4–20% pre-cast PAGE gels. Gels were transferred to polyvinylidene difluoride membrane (PVDF) membranes ((Bio Rad, Hercules, CA, USA) Immun-Blot with a pore size of 0.2 µm) via the Trans-blot Turbo System, and 5% BSA blocking buffer was added to incubate for one hour. Primary and secondary antibodies were diluted with TBS and 1% BSA. Primary antibody was used to probe for the desired protein and a secondary antibody is tagged with a fluorescent dye and binds to the primary antibody for detection. The primary antibody was exposed to the membranes overnight at 4 °C. These antibodies and their concentrations include β-actin (42 kD) 1:500 (Abcam, Cambridge, UK; ab115777), AT_1_ (42 kD), AT_2_ (40 kD) -1:500 (Alomone Labs, Jerusalem, Israel, AAR-011 and AAR-012), MAS1 (35 kD) 1:500 (InVitrogen, Waltham, CA, USA, PAS-PA5-43669), ATP1α_1_ (100 kD) 1:500 (ProteinTech, Wuhan, China), ATP1α_2_ (100 kD) 1:500 (ProteinTech, Wuhan, China), and Slc8a1 (120 kD) 1:500 (InVitrogen, Waltham, CA, USA). Membranes were viewed using the ChemiDoc Touch Imaging System (Bio Rad, Hercules, CA, USA) and a data analysis was completed using ImageLab software (version 6.1.0 build 7). All results were normalized to the housekeeping gene β-Actin. Seven biological replicates for HnSD and (mREN2)27 were represented on each gel and four technical replicates were analyzed on four gels. Each of the images was processed on the same membrane, one target at a time. To prevent the overlap of antibody targets that were in the same molecular weight, i.e., AT_1_, AT_2_, ATP1α_1_, and ATP1α_2_, a stripping process was employed between steps. The stripping was performed using Restore™ Western Blot Stripping Buffer (Thermo Fisher Scientific, Waltham, CA, USA) as per the manufacturer’s instructions. After each stripping, the removal of antibodies was verified by re-probing the membrane with a secondary antibody and confirming the absence of a signal. The membrane underwent blocking, and the rest of the process was carried out as described above. The representation of full membranes is presented in Supplementary Figure S2.

### 6.5. Isolation of RNA Nucleotides: qRT-PCR

Quantitative Reverse Transcription Polymerase Chain Reaction (PCR) measures mRNA content using the 5′ endonuclease activity of TaqDNA polymerase to cause the cleavage of an oligonucleotide probe and marked with a fluorescence label [19]. Rat heart total RNA was extracted using the RNeasy Mini Kit (Qiagen, Hilden, Germany). RNA quantification was performed using Qubit RNA high-sensitivity assay kits (Thermo Fisher Scientific, Waltham, CA, USA; Q32855). Total RNA, amounting to 10 ng, was transcribed to cDNA using the RT2 First Strand Kit (Qiagen, Hilden, Germany). For a specific target, RT-PCR was performed in a 25 μL reaction mixture consisting of 13 μL of carboxyrhodamine (ROX) qPCR Master Mix (Qiagen, Hilden, Germany), 1 μL of cDNA, and 0.3 μM of each primer. Real-time PCR amplification reactions were carried out using QuantStudio 3 real-time PCR systems (Applied Biosystems, Foster City, CA, USA) and qPCR design analysis software version 2.6.1 (Thermo Fisher Scientific) was utilized for analyzing the results. Rat-specific assay primers (Qiagen, Hilden, Germany) were used for β-actin (PPR06570C), AT_1_ (PPR44498A), AT_2_ (PPR57639F), MAS1 (PPR47225A), SERCA2 (PPR44845A), ATP α_1_(PPR66443B), and ATP α_2_ (PPR42386A). The qPCR primer assay was specific to angiotensin II receptor Type 1a (AT-1a) and not AT-1b. The data obtained were normalized to β-actin expression using the 2^−ΔΔCT^ method and expressed as a fold change compared with the control.

### 6.6. Statistical Analyses

The data are expressed as means ± SEM and ranges unless otherwise noted. Sample means were compared using paired or unpaired Student’s *t*-test statistics when only two variables were compared and differences between multiple means were analyzed for significance using a one-way analysis of variance (ANOVA) with a Tukey’s post hoc analysis. Simple linear regression was used to determine the relationship between various parameters of AngII-induced changes in the left ventricular myocytes. Means were considered to differ significantly if *p* values were <0.05. Statistical analyses were performed using GraphPad Prism 10.0.2(171).

## Data Availability

The original contributions presented in the study are included in the article/Supplementary Materials, further inquiries can be directed to the corresponding authors.

## Supplementary Materials

The following supporting information can be downloaded at: https://www.mdpi.com/article/10.3390/ijms25136997/s1.
