# Supplementary material for: Impact of RAAS Receptors and Membrane-Bound Transporter System in the Left Ventricle during the Long-Term Control of Hypertension
Source: Int J Mol Sci. 2024 Jun 26;25(13):6997. doi: 10.3390/ijms25136997 (PMC11241669; doi:10.3390/ijms25136997)
Supplement: Supplementary file 1 [file ijms-25-06997-s001.zip › ijms-3033368-supplementary.pdf]

*Supplement*

# Impact of RAAS receptors and Membrane-Bound Transporter System in the Left ventricle during the Long-Term Control of Hypertension

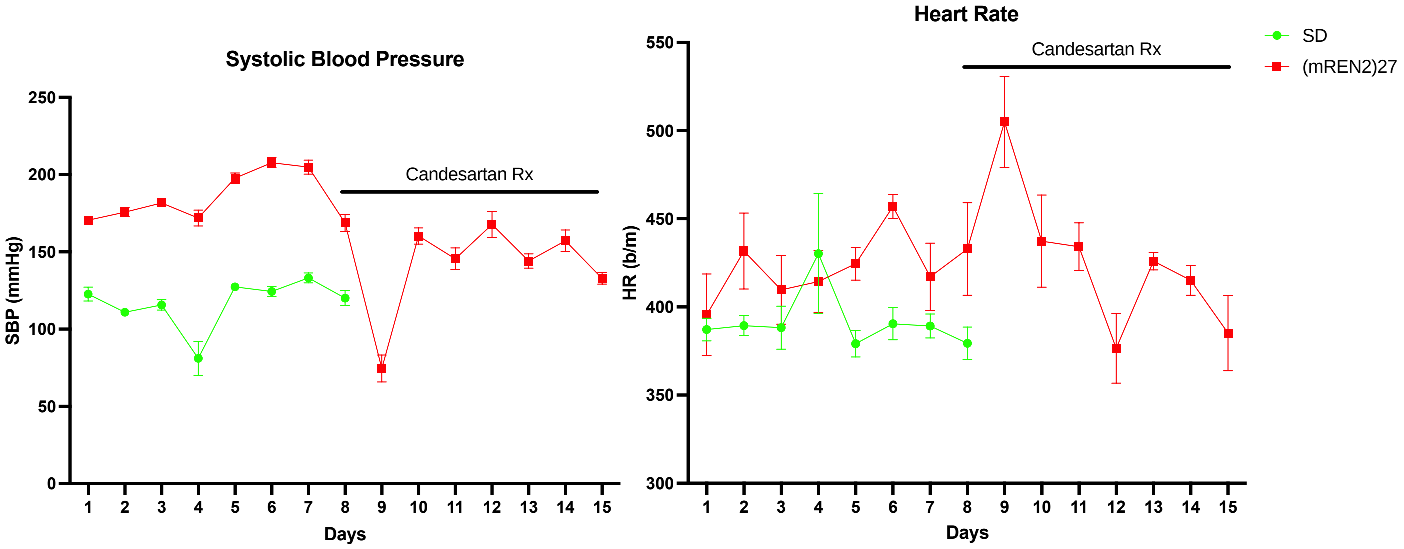


**Figure S1:** Systolic Blood Pressure (SBP) and Heart Rate (HR) over a 15-day period with and without Candesartan treatment. The graph shows SBP and HR measurements of Hannover Sprague Dawley (HnSD), (mREN2)27, and (mREN2)27 treated with the angiotensin receptor blocker (ARB), Candesartan (CandRx). CandRx lowered blood pressure and normalized resting heart rate, suggesting AngII effects on heart rhythmicity via the $AT_1$ receptor subtype.

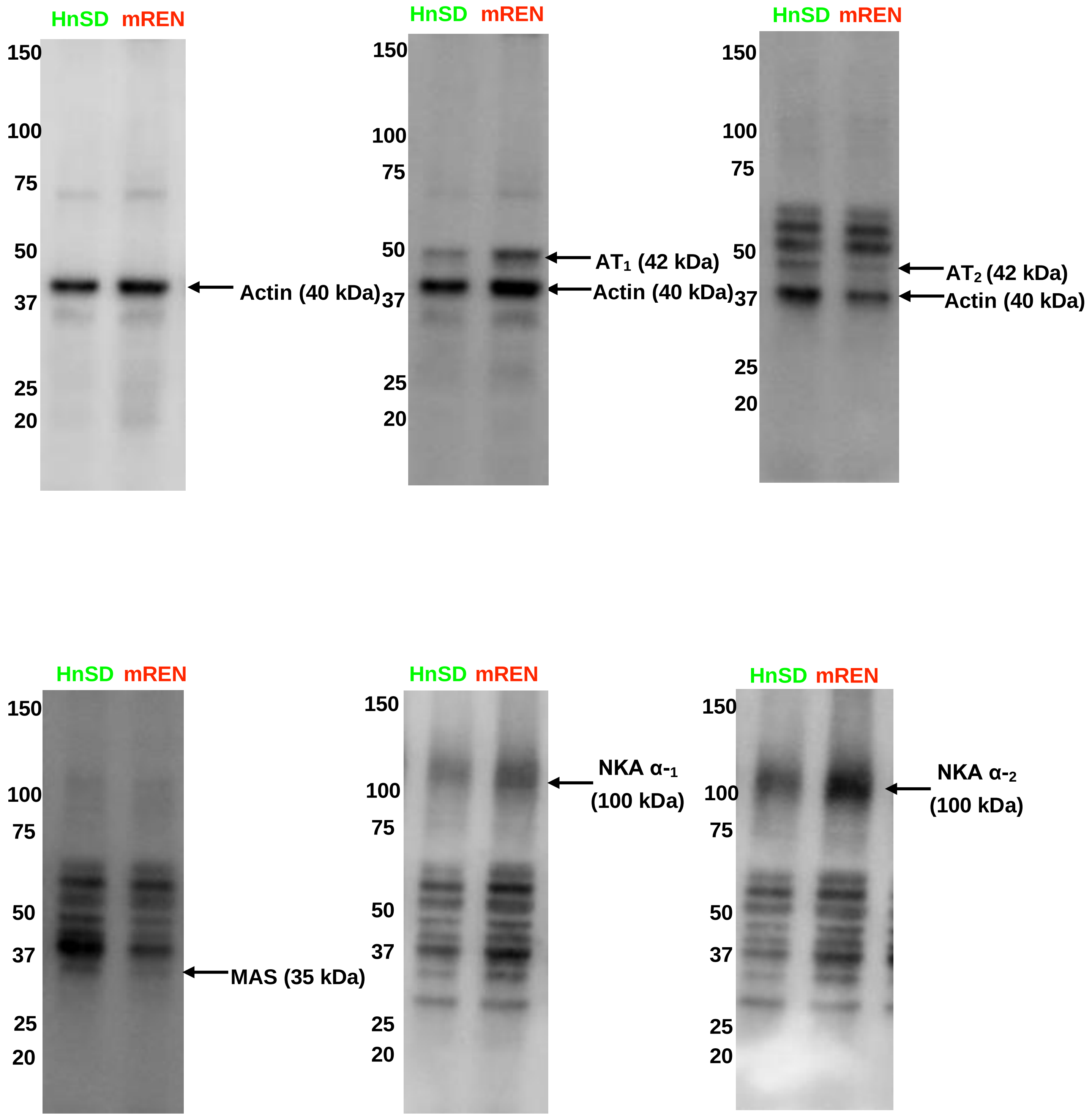


**Figure S2**: Representation of full membranes A) Actin (40 kDa), B) $AT_1$ (42 kDa), C) $AT_2$ (40 kDa), D) MAS (35 kDa), E) NKA $\alpha$-$_1$ (100 kDa) and F) NKA $\alpha$-$_2$ (100 kDa).
